# Finding parasites and finding challenges: improved diagnostic access and trends in reported malaria and anti-malarial drug use in Livingstone district, Zambia

**DOI:** 10.1186/1475-2875-11-341

**Published:** 2012-10-08

**Authors:** Freddie Masaninga, Masela Sekeseke-Chinyama, Thindo Malambo, Hawela Moonga, Olusegun Babaniyi, Helen Counihan, David Bell

**Affiliations:** 1 World Health Organization country office, Lusaka, Zambia; 2 Malaria Consortium Office, Lusaka, Zambia; 3 Livingstone District Health Management Team, Livingstone, Zambia; 4 National Malaria Control Centre, Ministry of Health, Lusaka, Zambia; 5 Malaria Consortium Office, London, UK; 6 Foundation for Innovative New Diagnostics (FIND), Geneva, Switzerland

## Abstract

**Background:**

Understanding the impact of malaria rapid diagnostic test (RDT) use on management of acute febrile disease at a community level, and on the consumption of anti-malarial medicines, is critical to the planning and success of scale-up to universal parasite-based diagnosis by health systems in malaria-endemic countries.

**Methods:**

A retrospective study of district-wide community-level RDT introduction was conducted in Livingstone District, Zambia, to assess the impact of this programmed on malaria reporting, incidence of mortality and on district anti-malarial consumption.

**Results:**

Reported malaria declined from 12,186 cases in the quarter prior to RDT introduction in 2007 to an average of 12.25 confirmed and 294 unconfirmed malaria cases per quarter over the year to September 2009. Reported malaria-like fever also declined, with only 4,381 RDTs being consumed per quarter over the same year. Reported malaria mortality declined to zero in the year to September 2009, and all-cause mortality declined. Consumption of artemisinin-based combination therapy (ACT) dropped dramatically, but remained above reported malaria, declining from 12,550 courses dispensed by the district office in the quarter prior to RDT implementation to an average of 822 per quarter over the last year. Quinine consumption in health centres also declined, with the district office ceasing to supply due to low usage, but requests for sulphadoxine-pyrimethamine (SP) rose to well above previous levels, suggesting substitution of ACT with this drug in RDT-negative cases.

**Conclusions:**

RDT introduction led to a large decline in reported malaria cases and in ACT consumption in Livingstone district. Reported malaria mortality declined to zero, indicating safety of the new diagnostic regime, although adherence and/or use of RDTs was still incomplete. However, a deficiency is apparent in management of non-malarial fever, with inappropriate use of a low-cost single dose drug, SP, replacing ACT. While large gains have been achieved, the full potential of RDTs will only be realized when strategies can be put in place to better manage RDT-negative cases.

## Background

To achieve universal access to parasite-based diagnosis of malaria-like febrile illness, consistent with current global policy recommendations [1], it will be necessary to deploy malaria rapid diagnostic tests (RDTs) widely at community level in most malaria endemic countries. Microscopy services, the only realistic alternative in low resource settings, cannot be sustained at this level in most programmes [2], and, therefore, over 100 million RDTs were financed towards filling this gap in 2010 [3]. While it is feasible to train community-level health workers to safely prepare and accurately interpret RDTs [4-6], and evidence exists that withholding treatment on the basis of a negative RDT results can be safe [7-13], RDT introduction will be pointless if the diagnostic outcome does not guide subsequent case management. This should include both restriction of anti-malarial drugs to parasite-positive cases, and appropriate early management of non-malarial febrile illness.

Artemisinin-based combination therapy (ACT), the first line anti-malarial for *Plasmodium falciparum* infection in most endemic countries, is relatively expensive and reliant on an insecure supply of raw materials. Meanwhile, non-malarial febrile illness exerts a higher childhood mortality across malaria-endemic countries than malaria. [14]. RDTs, therefore have an important role to play in targeting ACT prescription and identifying non-malarial fever for other management. However, reports of the impact of RDT results on subsequent management vary. While impact on anti-malarial consumption on a wide-scale is demonstrated [15-17], a number of published field trials have also revealed poor adherence to results, likely to reduce or negate the potential of RDTs to improve disease management [18-21].

Previously in Zambia, malaria diagnosis had been predominantly based on symptoms, with only 12.5% of 1730 public health facilities listed as having diagnostic capacity by microscopy in 2004 [22]. The Ministry of Health revised national guidelines in 2009-2010 to mandate parasitological confirmation of malaria based on microscopy or RDTs in the public and private sectors, with RDTs to be used where microscopy was not available, or where RDT use on an out-patient basis by non-laboratory staff would reduce laboratory workloads [23]. This policy built on a decision of the National Malaria Control Centre (NMCC) to progress to national scale-up of RDT use in 2009, after a number of RDT trials and introduction of malaria RDTs in certain districts in the public sector [15,24,25]. ACT had been introduced as first-line therapy in 2005.

As part of this trial of RDTs at community level, a study was conducted in Livingstone District to test and improve RDT instructions and training materials, and trained community health workers (CHWs) in the district were followed to assess performance in RDT use over 12 months [6]. In view of the dramatic reduction in reported malaria at the district level, resulting in a decline from first to 16^th^ in the District Health Management Team’s (DHMT) list of health priorities (DHMT, Personal communication), a retrospective study was undertaken to review the reported malaria rate, mortality, and consumption of anti-malarial drugs in Livingstone District across the study period, and assess the impact of RDT introduction on these.

## Methods

### Geographical area

**Figure 1:**
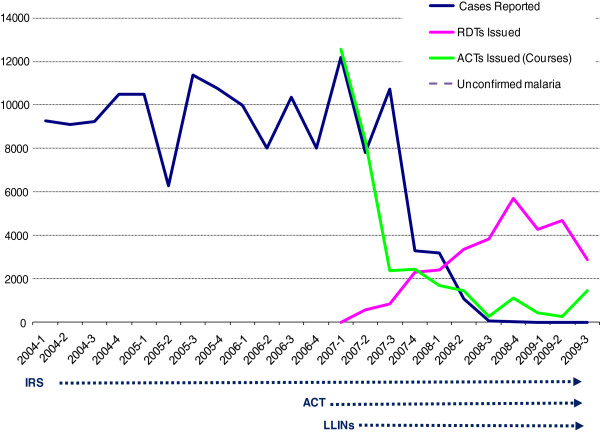
**Quarterly reported malaria incidence in HMIS, and ACT and RDT dispensing to clinics from the District Office, Livingstone District, Zambia.** Date of commencement of anti-malarial interventions in Livingstone District are illustrated: Indoor-residual spraying in first quarter 2004, Artemisinin-based combination therapy (ACT) as first-line therapy in fourth quarter 2006, and long-lasting insecticide-impregnated bednets (LLINs) in first quarter 2007.

Livingstone district is located in southern Zambia, bordering Zimbabwe. The district is relatively small in area by Zambian standards, covering 672 square kilometres [26], with a population of 142,034. The economy is based mainly on agriculture and the tourist industry based around Victoria Falls. The climate includes a wet season extending from October to April-May and a dry season from May to September. The Livingstone DHMT oversees 13 health centres and approximately 65 community health workers, the latter overseen locally by the nearest clinic. Malaria had previously been considered the primary public health priority in Livingstone district, with between 6,000 and 12000 thousand cases reported in each quarter during 2004 to 2006 inclusive, based on symptom-based diagnosis (Figure 1). The limited microscopy-based diagnosis in place at some health centres reported 98% *P. falciparum*[27]. Indoor residual spraying (IRS) was introduced in Livingstone district in 2004, insecticide-treated bed nets in early 2007, and the first-line anti-malarial treatment changed from sulphadoxine-pyrimethamine (SP) to artemisinin-based combination therapy (ACT) using artemether-lumefantrine in late 2006, in line with Zambian national policy. Quinine continued to be recommended as second-line therapy, and SP was supplied for intermittent preventive treatment in pregnancy (IPTp) and for malaria treatment in patients less than 5 kgs. RDTs had not previously been in routine use in Livingstone District or used by community health workers, but had been introduced to the District Hospital and some clinics from 2005 on a limited basis. Microscopy use is restricted to the district hospital and four health centres and contributes little to total malaria diagnostic figures.

### Health centres and health staff

In November and December 2007, 65 CHWs of Livingstone District, Zambia, were trained in RDT use, as part of a national roll-out of home management of malaria (HMM). This training was linked to a study to develop and test improved instruction and training materials [4]. The 13 clinic staff, and most (51) CHWs had prior experience in malaria management, though mainly in symptom-based treatment as only two had previously used malaria rapid tests. Full characteristics of the CHWs are described elsewhere [6]. All received 3.5 days training in case management, including a half day specifically devoted to preparation and interpretation of *P. falciparum*-detecting malaria RDTs (ICT Malaria Pf, *ICT Diagnostics*, South Africa), and were provided with product-specific RDT job-aids and interpretation guides [28], RDTs and ACT (artemether-lumefantrine, [Coartem®]). They were instructed to test febrile patients and treat with ACT only if the RDT result was positive, and refer cases of significant fever but negative RDT results. CHWs were followed with routine supervision and specific observational review at 3, 6 and 12 months to assess blood safety and diagnostic performance [6].

CHWs were requested to maintain records of RDTs used, results, and ACT dispensed in a record book and report these to the local clinic. RDT negative fever cases, apparent malaria treatment failures, severe infections and malaria-negative patients were also to be referred to the nearest health clinic, in line with national guidelines [23]. Health centres are provided with oral and parenteral quinine for severe malaria, early pregnancy and first-line treatment failures, and SP for intermittent prophylactic treatment for pregnancy (IPTp). Clinics stock antibiotics and anti-pyretics for non-malarial fever.

CHWs were supervised by the local clinics according to normal practice and resupplied from clinic stock. Health centres, staffed by trained nurses, received RDT and anti-malarial drug stock from the DHMT, and were also trained by the DHMT to use RDTs and institute parasite-based malaria diagnosis. During 2008 it was observed that some health centre staff continued to base malaria treatment only on clinical diagnosis, and the DHMT re-trained all health centre staff on fever case management and use of RDTs from April to May 2008, with increased emphasis on rational use of ACT.

### Incidence and consumption data

Malaria incidence and mortality data used for this study are from data submitted by the Livingstone DHMT to the Zambian National Health Information Management System (HMIS), from the first quarter of 2004 to the third of 2009. These data are derived from clinic reports which are reviewed and data checked prior to submission to the DHMT by the health centre ‘in-charges’. The reporting period for commodity use for this study extended from January 2007 to October 2009, the first year being part of a study of quality of health worker RDT performance described elsewhere [6]. During November and December 2009, personnel from the NMCC and Malaria Consortium (MC) collated information on quantities of ACT, SP, Quinine and RDTs dispensed by the district office from data in the district stock control cards, while information on quantities of these supplies received by the health centres was obtained from the health centre stock control cards, supply vouchers and health centre RDT registers. Morbidity and mortality statistics were subsequently obtained from the DHMT and district hospital, including a review of some malaria mortality case notes to determine whether diagnosis was confirmed. Data are presented quarterly, to smooth variations in commodity requests and dispensing.

Consumption and dispensing data and malaria reporting were also reviewed at clinic and CHW level by visiting each of the 13 health centres and 45 CHWs who could be located at home and visited during the eight day data review. Stock control cards, RDT registers and case record books were reviewed and data recorded for the period since introduction of HMM to the point of data collection (November 2007 to September 2009). In all cases, records were incomplete, including periods of non-reporting of all data in the majority of cases, absent data in some reporting categories, and inconsistencies incomplete reporting. There were no records at some health centres to indicate supplies (RDTs, ACT, SP or QNN) received and used, most stock control cards at health centres were not updated, RDTs registers that were not in use had often been discarded. In several clinics, stock cards were absent, while several clinics shared a single case record book between several clinicians, resulting in incomplete data entry. Data obtained from the CHW was at times not integrated in the health centre data, and the supply of RDTs to the CHWs was incompletely documented by health centres. After review of data, CHW and clinic data were, therefore, excluded from analysis.

Data on overall malaria test positive rates was obtained directly from DHMT records for 2009 and 2010, obtained after the main study period. A number of deaths reported as malaria in 2009 in Livingstone District Hospital figures were investigated through a specific review of the medical records by health staff to determine true malaria diagnostic status, i.e. to see if there was a record of microscopy or an RDT being performed.

### Analysis

Data were entered in MS Excel directly from stock records, and analysis performed using the same software. In view of the relatively short time-period of the observed intervention, reliance on central consumption data that does not directly reflect actual patient consumption at that time, and the fluctuating nature of previously reported malaria incidence, trend analysis has not been attempted and the data are presented as direct comparisons between time periods.

### Institutional approval

The study was conducted as part of the National Malaria Control Centre-WHO collaborative work to generate data needs (evidence base) for the NMCC in Zambia. The underlying study on RDT job-aid development received ethical approval through WHO/TDR and the Tropical Disease Research Centre, Ndola, Zambia [6]. As this study reviewed only aggregated retrospective health service data, no further approval was required.

## Results

Reported malaria in Livingstone district (cases recorded in the District Health Office records derived from routine HMIS data) remained above 8,000 cases per quarter (three-month period) from 2004 until the third quarter of 2007, with minor declines in the third quarters of 2005 and 2007. Reported incidence then rapidly declined to only 65 cases reported in the third quarter of 2008, and an average of 12.25 cases per quarter over the year to September 2009 (Figure 1). The decline coincided with the introduction of malaria RDTs, though its magnitude was greater than the total number of RDTs dispensed (an average of 4,381 per quarter in the year to September 2009). From the second quarter of 2008, a new category of ‘confirmed and unconfirmed malaria’ was reported separately in health statistics over this period. ‘Malaria’, restricted to laboratory confirmed malaria (RDT or microscopy) continued to decrease, with only two cases reported in the third quarter of 2009. However, ‘confirmed and unconfirmed’ malaria rose, with the difference (i.e. unconfirmed cases) rising from 177 to 378 per quarter (an average of 294 unconfirmed malaria over the last 12 months). Taking data from the first three quarters of years 2007 and 2009 from which comparative data is available, therefore taking seasonal variation into account, malaria declined from 30,723 cases in 2009 to 23 confirmed and 1022 total cases (including new ‘unconfirmed’ category), a reduction of over 99.9% and 96.6% respectively.

**Figure 2:**
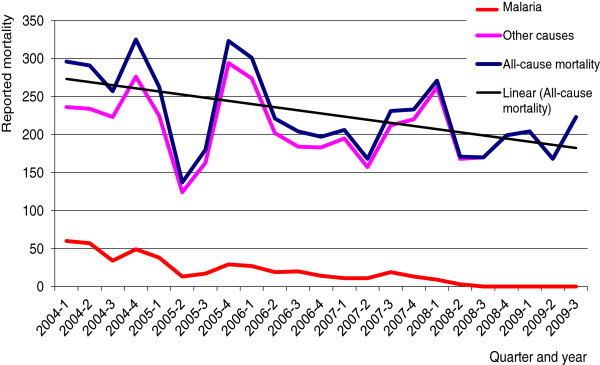
Quarterly malaria and all-cause mortality reported in HMIS data in Livingstone District, Zambia.

Reported malaria mortality declined from 60 cases in the first quarter of 2004 to zero cases in the third quarter of 2008, and remained at zero through the first three quarters of 2009 (to the end of the study period). Confirmed malaria deaths remained at zero through 2009 (Figure 2). All-cause mortality dropped over the same period from 296 in the first quarter of 2004 to 223 in the third quarter of 2009, but with considerable fluctuations (R^2^ = 0.27). Record tracing of 11 deaths reported as fully or partially attributed to malaria in district health statistics in 2009 found all to have been unconfirmed (untested by RDT or microscopy).

RDT dispensing from the District office commenced in the second quarter of 2007, and increased rapidly from the last quarter of that year coinciding with the training of CHWs in community-based diagnosis and management of malaria (Table 1). After a peak of 5,700 RDTs dispensed in the last quarter of 2008, a decline in dispensing followed to 2,875 at the end of the study period (third quarter of 2009), with an average of 4381 per quarter over the last 4 quarters.

**Table 1:** Rate of issue of RDTs and ACT from district level to health centres from start of 2007 to late 2009 in Livingstone District, Zambia, and recorded malaria rate for the corresponding period

**Time period**	**RDTs issued**	**ACTs issued (Courses)**	**Recorded malaria**^**a**^	**‘Unconfirmed’ malaria**
1st QTR-2007	0	12550	12186	
2nd QTR-2007	575	8340	7806	
3rd QTR-2007	850	2358	10731	
4th QTR-2007	2300	2447	3295	
1st QTR-2008	2400	1694	3196	
2nd QTR-2008	3350	1470	1081	
3rd QTR-2008	3850	270	68	
4th QTR-2008	5700	1128	26	177
1st QTR-2009	4275	450	8	276
2nd QTR-2009	4675	270	13	345
3rd QTR-2009	2875	1440	2	378

a: From the 4^th^ quarter of 2008, this included only laboratory confirmed malaria (i.e. RDT or microscopy).

**Figure 3:**
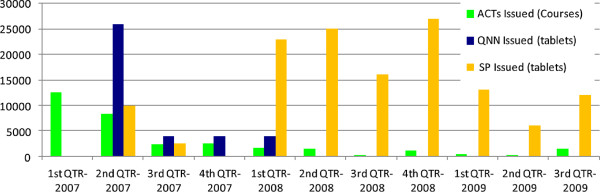
**Anti-malarial drugs dispensed from the District office pharmacy to health facilities in Livingstone District, 2007-2009.** ACT is dispensed as full treatment courses, while quinine and SP (sulphadoxine-pyrimethamine) are dispensed and recorded as single tablets. 30 Quinine and 3 S-P tablets equate to a typical full adult course.

ACT consumption (based on stocks released from the District pharmacy) declined rapidly from early 2007, remaining well below initial levels and below RDT consumption from late 2007 onward after the health worker training (Figure 3). The initial decrease was of greater magnitude than the increase in RDT use over the same period. Comparing the first 3 quarters of 2007 and 2009, consumption declined from 23248 courses to 2160 (>90%) across the same seasons. ACT use remained well above the reported malaria rate, however, with 1,440 courses dispensed to clinics in the last quarter of 2009 and a mean of 822 per quarter over the last 12 months of the study (Table 1 and Figure 3).

Quinine was not reported to be dispensed from the District pharmacy in the first quarter of 2007, but after 26,000 tablets were dispensed in the second quarter, only 4,000 were dispensed in the subsequent quarters and no quinine was sent to clinics after early 2008 (Figure 3). In contrast, dispensing of SP from the District office rose after RDT introduction, with 12,600 doses dispensed throughout 2007 and 88,000 in 2008 (equivalent to 4,200 and 26,000 adult courses respectively), remaining relatively high up to the end of the study period (Figure 3).

While the RDT positive rate is not well documented in available district data over the years of the study on a case by case basis, it is possible to see trends by comparing data from the study period to more recent data. DHMT data from 2009 records 3,605 suspected cases tests (RDTs or microscopy) and 119 positive results, while in 2011 2,926 suspected malaria cases were tested and 72 were positive.

## Discussion

This study indicates that the introduction of malaria RDTs into Livingstone District in southern Zambia was associated with a large reduction on reported malaria and large changes in anti-malarial drug consumption. It also illustrates the challenges faced by health services in dealing with the fundamental changes in febrile disease management that the introduction of parasite-based diagnosis of malaria requires, and the gaps in health system data that can complicate their understanding and management.

While the reduction in reported malaria in Livingstone district is closely associated with the advent of parasite-based diagnosis it was of far greater magnitude than the eventual rate of use of RDTs. Thus, while RDTs contributed to reduced malaria reporting, other factors are also at play. Clinicians were probably imposing stricter definitions of malaria-like fever before RDTs arrived. From late 2008, the reported malaria rate in Figure 1 must be largely accounted for by exclusion of parasite-negative cases by RDT. No RDT stock-outs occurred at a district level during this time (NMCC, Personal communication). Despite the uncertainty in previously-reported malaria mortality, given the apparent uncertainly of previous malaria prevalence overall, the elimination of reported malaria mortality indicates that this tightening of the definition of malaria-like fever and restriction in RDT use and subsequent ACT consumption did not result in significant misdiagnosis of malaria and progression to severe disease.

It is not yet possible to determine whether malaria is declining in Livingstone District, or whether malaria has been uncommon for several years and the use of RDTs has made this visible. It is reasonable to assume that the preceding interventions (IRS and bed net introduction, and change of first-line anti-malarial therapy to an ACT) have had an impact (Figure 1), and indications of reduction in real malaria incidence are noted elsewhere in southern Zambia [29,30]. Whatever the reasons, the reduction in reported malaria in district health statistics, and very low confirmed malaria rate among those tested, will enable a more effective allocation of health resources to other areas of need; malaria has dropped from first to sixteenth in the district’s disease incidence statistics (Personal Communication; DHMT) and malaria mortality was rare or eliminated at the end of the study period. This is a remarkable change in perceptions of local health priorities.

While ACT consumption declined to very low levels after RDT introduction compared to previous dispensing rates, consumption of ACT remained above the reported (now confirmed) malaria incidence, suggesting a leakage to non-malaria cases or incomplete case reporting (Figure 1). However, large reductions in ACT (and quinine) use clearly resulted. These have potential financial benefits for the programmed. A total of 7,065 packs of ACT were returned to the Zambian Central Medical Store unused from Livingstone District in February 2009, and a further 157 expired at the end of 2009 at the District pharmacy (Personal communication, DHMT).

Despite the major decline in ACT consumption, it continued to be consumed at levels above the combined confirmed and unconfirmed malaria rate. Also significant is the rise in the consumption of the other available anti-malarial; SP. The full potential health benefit on reduced anti-malarial consumption was therefore not being realized. Reasons may include pressure from patients for treatment for what they traditionally regard as ‘malaria’, lack of alternatives (or a need to educate health providers and patients on the appropriateness of alternatives), and low cost of the drug (little financial penalty to the health system). Health providers may also be reluctant to completely reverse years of accepted wisdom that ‘fever equals malaria’. Incomplete adherence to RDT results is well described elsewhere, [18-21,25], but appears far less of a problem here. Well-directed training messages to health workers and the community may have played some role in this, as health workers were specifically warned to expect most RDT results to be negative, and that malaria was considered highly over-diagnosed. The outcomes are also consistent with observations elsewhere of a lag followed by eventual good adherence when RDTs are implemented at a large scale [15,16].

In order to fully understand the pattern of consumption of anti-malarial drugs (ACT, SP and QNN), their relationship to RDT use, and to devise new management strategies, it will be important to gather better information from the point of care. The reporting of unconfirmed malaria, added to district statistics in late 2008, may indicate local RDT stock-outs, or some incomplete trust in, or adherence to, RDT results. The data reflects receipt and action on stock requests, not actual dispensing and consumption at a patient level.

No information was obtained on consumption of anti-malarial medicines in the private sector; it is possible that some patients denied ACT after a public sector diagnosis may have sought medicine through this route. Such issues are common in such settings where limited resources and high staff workloads combine, and will require innovative approaches to ensure accurate and timely reporting [31,32]. Zambia commenced introduction of a new software-based health management information system (HMIS) soon after the end of the study period and it is hoped this will help to address this area of need.

Despite incomplete adherence, this sea-change in patient management through parasite-based diagnosis revealed a previously hidden majority of misdiagnosed febrile patients. This is an essential step toward the development of a more appropriate management strategy to deal with the syndrome that is acute febrile disease. Current efforts to integrate management of acute respiratory and gastro-intestinal infection into community case management of malaria are a step along this path [33]. For Livingstone District, and other areas with similar declines in reported malaria, two major challenges arise. Firstly, management of non-malarial febrile illness is hampered by poor diagnostic capacity and limited availability of treatment; hence the increase in use of SP in the study area. Success in addressing fever as a syndrome rather than just treating malaria will remain elusive as long as most available infectious disease implementation funding for low-resource countries is directed toward only three diseases; malaria, tuberculosis and HIV, and funding for research and development is similarly restricted [34]. Secondly, interest in malaria must be maintained as its incidence declines below that of other severe illnesses. Long-lasting bed nets will require replacement after three to five years, IRS may require continued annual campaigns, and the availability of RDTs and ACT must be maintained to detect and treat the few remaining malaria cases. New ways of managing malaria, as an uncommon disease but potential public health emergency, will need to be developed and resources appropriately targeted.

## Conclusion

The introduction of parasite-based community-level diagnosis of fever in Livingstone District has demonstrated a strong association between introduction of malaria RDTs and reduction in inappropriate use of ACT. A corresponding decline of reported malaria mortality to zero suggests high safety of RDT-based diagnosis in this population. However, the full potential impact of RDT introduction has not been reached due to continued treatment well above the confirmed malaria rate, for reasons not clear under the health information system in place at the time. The results also demonstrate the importance of developing appropriate strategies for management of the multiple causes of non-malarial fever. Inappropriate consumption of ACT may be replaced by inappropriate consumption of other drugs if the next step is not taken to manage fever as a syndrome, rather than as a case requiring only a malaria diagnostic test and potentially anti-malarial drugs.

## Competing interests

The authors declare that they have not competing interests.

## Authors’ contributions

FM participated in the implementation of the study and provided inputs into the manuscript. M S-C participated in the design and led the implementation of the study. TM participated in the implementation of the study. HM participated in the implementation of the study. OB participated in the coordination of the study. HC participated in the design and coordination of the study. DB conceived of the study, participated in its design and drafted the manuscript. All authors read and approved the final manuscript.
